# Serological Investigations of Bluetongue Virus (BTV) among Sheep and Goats in Kassala State, Eastern Sudan

**DOI:** 10.1155/2020/8863971

**Published:** 2020-10-01

**Authors:** Molhima M. Elmahi, Abdel Rahim E. Karrar, Amira M. Elhassan, Mohammed O. Hussien, Khalid A. Enan, Mohammed A. Mansour, Abdel Rahim M. El Hussein

**Affiliations:** ^1^Kassala Veterinary Research Laboratory, Animal Resources Research Corporation (ARRC), P.O. Box 237, Khartoum, Sudan; ^2^Faculty of Veterinary Medicine, University of Khartoum, Ministry of Higher Education and Scientific Research, P.O. Box 32 Khartoum North, Khartoum, Sudan; ^3^Central Veterinary Research Laboratory (CVRL), Animal Resources Research Corporation (ARRC), P.O. Box 8067 El Amarat, Khartoum, Sudan; ^4^Central Laboratory, Ministry of Higher Education and Scientific Research, P.O. Box 7099, Khartoum, Sudan

## Abstract

Bluetongue (BT) is an infectious, noncontagious, vector-borne viral disease of wild and domestic ruminants. BTV is a member of the *Orbivirus* genus of the family Reoviridae. The present study aimed to investigate the seroprevalence of BTV in sheep and goats in Kassala State, Sudan. It also aimed to determine risk factors associated with BTV infection. The study was carried out by a structured questionnaire survey, and a total of 809 serum samples were collected from sheep (*n *= 459) and goats (*n *= 350) from 9 different localities in Kassala state. These samples were analyzed using a competitive enzyme-linked immunosorbent assay (cELISA) for the detection of BTV antibodies. The overall seroprevalence of BTV was 91.2% (738/809). In goats, the prevalence of BTV antibodies was comparatively higher (100%) than in sheep (84.5%). The prevalence differed between localities and was the highest in the center section of Kassala and Western Kassala (100%). Animals aged 6–11 months were highly infected (93.9%) compared to 1-year-old (85.5%). Caprine species was more likely to be infected (100%) than ovine (84.5%), and females were highly infected (92.8%) than males (85.5%). BTV infections were higher in the winter season (91.4%). Risk factors that showed significant associations with cELISA positivity included locality and sex (*p* ≤ 0.003) and species and age (*p* ≤ 0.000). Factors significantly associated with cELISA positivity in multivariate analysis were localities, species, age, and sex. BTV infection is prevalent in sheep and goat populations in Kassala state.

## 1. Introduction

Bluetongue (BT) is an infectious, noncontagious, arboviral disease that affects wild and domestic ruminants including sheep, goats, cattle, buffaloes, deer, most species of African antelope, and various other Artiodactyla [1]. BTV belongs to the *Orbivirus* genus of the family Reoviridae [2]. Bluetongue infection in sheep and goats may present as an acute, chronic, or subclinical disease; the acute disease is characterized by fever, facial edema, hemorrhages, and ulcerations on the oral mucosa and coronitis [3, 4]. In cattle, the disease is mostly subclinical [5] except in the case of BTV-8 infection in which clinical signs are shown by a large number of animals [6]. Bluetongue virus (BTV) is transmitted by biting midges (*Culicoides* spp.) [7]. Economic losses due to BT disease can be direct or indirect. Direct losses are incurred by losses in production (due to mortality, abortion, and reduced production of milk and meat), cost of vaccines, and control expenditure, while indirect losses are attributed to the lost revenue due to trade restrictions that limit access to higher value markets [8]. Until today, 27 serotypes of this virus have been reported worldwide; however, there are further putative serotypes that have been reported recently [9, 10]. Serogroup-specific antibodies against BTV can be detected by a competitive ELISA test that detects the VP7 protein [11]. There are several commercial ELISA kits that have been developed to detect early antibodies against BTV in individual or bulk milk samples [12]. In addition, BTV antibodies can be detected using serum neutralization test (SNT) which is the most serotype-specific and sensitive of all the tests but is the costliest and time-consuming test [13, 14]. BTV can be isolated by propagation in embryonated chicken eggs, in cell cultures, or in susceptible animals [15].

BTV genome can be detected by real-time PCR which is rapid and highly sensitive in the nucleic acid samples that originate from blood or tissues. Serogroup-specific RT-PCR assays for BTV, targeting the more conserved regions of the virus genome (e.g., Seg-1, 5, 7, and 10), have been developed and successfully tested [16–25].

In Sudan, BT disease was first reported in the country in 1953 when an outbreak took place in sheep in the Gezira research farm in Wad Madani in Central Sudan [26]. Subsequently, serological surveys indicated that BTV antibodies were widespread in the domestic species of livestock including sheep, goats, cattle, and camels in the country [27–29]. Later on, the virus was isolated from outbreaks of sheep, from apparently healthy cattle, and from *Culicoides* midges [30–32], and five BTV serotypes were reported up to now, serotypes 1, 2, 4, 5, and 16, which are endemic in various states of Sudan [33, 34].

Fayza et al. [35] explained the role of apparently healthy cattle as a reservoir of BTV to sheep in Sudan, and Elzein [31] reported that susceptibility of camels to BTV infection was lower than that of other ruminant species in the country. Elhassan et al. [36] showed that BTV antibodies in cattle were quite prevalent in Gezira (Central Sudan) and were particularly high in older animals, females, cases of infertility, and during rainy season.

The presence of BT disease in Kassala state in Eastern Sudan has long been suspected. Our current study has thus been carried out to investigate BTV seroprevalence and to determine risk factors associated with BTV infection in sheep and goats in this animal resource-rich part of the country.

## 2. Materials and Methods

### 2.1. Study Area

**Figure 1:**
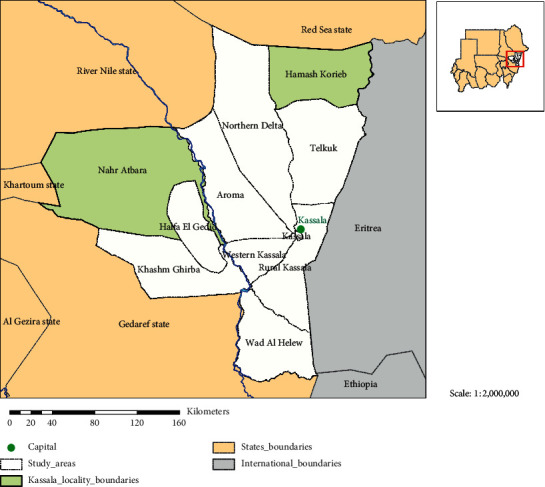
Map of Kassala state showing the locations where tissue specimens and sera samples were collected. The study area is located between longitude 14–17° N and latitude 34–37° E.

The survey was carried out during the period of June, 2015 to March, 2016 in 9 localities in the Northern, Southern, Eastern, and Western Kassala state. The investigation area lies between latitudes 14˚N and 17˚N and longitudes 34˚E and 37˚E. This area is a poor savanna in the north and east and rich savanna in the south and west. A map of Kassala state representing different localities is shown in Figure 1.

### 2.2. Study Design

The present investigation is a cross-sectional survey in Kassala state of Eastern Sudan. A multistage probability sampling method was used in this study. Nine localities in Kassala state were selected randomly. Samples were collected from apparently healthy sheep and goats from the north (Aroma and Northern Delta localities), south (Wad Al Helew and Khashm Ghirba localities), east (Rural Kassala locality), central section (Kassala locality), northeast section (Telkuk locality), and west section (Western Kassala and Halfa localities). Sample size for the study was estimated using the formula *n* = *z*2*PQ*/*L*2 [37], where *n* is the required number of individuals to be examined; *z* is a constant = 1.96; *P* is known or estimated prevalence; *Q* = (1−*P*); and *L* is the allowable error. The estimated animals' number using this formula assuming 50% prevalence rate was 384. A total number of 809 serum samples that were collected randomly from sheep and goats (459 sheep and 350 goats) in 9 localities were included in this study.

### 2.3. Questionnaire

A structured questionnaire survey was applied to all animals recruited in the study. Information pertaining to the survey was obtained from the animal owners. Individual and management risk factor attributes were included in the questionnaire. The risk factors included species (caprine and ovine), sex (male and female), age (6–11 months and 1 year and above), and season (rainy (July–November), winter (December–February), and summer (March–June)). In addition, the section to which the nine localities belong was also included in the study.

### 2.4. Collection of Blood Samples

Blood samples were collected from the jugular vein of individual sheep and goats in plain Vacutainer tubes by well-trained veterinarians. In total, 809 blood samples were collected randomly from the animals and used in this study. Blood samples were allowed to clot, and sera were collected and kept frozen at −20°C until used for screening BTV antibodies.

### 2.5. Enzyme-Linked Immunosorbent Assay (ELISA)

Samples were tested using bluetongue competition antibody test ELISA Kits (IDEXX, USA) to detect group-specific BTV antibodies. The procedure was conducted according to the manufacturer's instructions. The tested sera were considered positive when they produced an optical density less than or equal to 70% of the mean of the negative controls (S/N). The tested sera that produced an optical density greater than or equal 80% of the mean of the negative controls (S/N) were considered negative, and the tested sera that produced an optical density greater than 70% and less than 80% of the mean of the negative controls were considered doubtful and were retested.

### 2.6. Statistical Analysis

The results of serology and other information collected during this investigation such as locality, section, season, sex, breed, age, and clinical signs were compiled and managed using descriptive analysis. The statistical computation was done using statistical package SPSS version 20 (SPSS Inc., Chicago, USA). To identify the associations of the risk factors with the specific viral seroprevalence, the chi-square test (*x*^2^ test) was employed. The statistical significance level was set at *p* ≤ 0.05.

## 3. Results

Using cELISA, the overall seroprevalence of BTV-specific IgG was 91.2% (738/809) in sheep and goats. The prevalence in sheep was 84.5% (388/459), and in goats, it was 100% (350/350). The prevalence of BTV antibodies was 91.5% (238/260) in the east section of Kassala state; 96.5% (139/144) in the west section of the state; 90.7% (146/161) in the north section; 92.9% (52/56) in the south section; 84.5% (136/161) in the northeast; and 100% (27/27) in the center (Table 1). Regarding localities, the highest rates of BTV seropositivity in sheep were recorded in the center section of Kassala and Western Kassala locality in the west section of the state (100%), whereas the lowest rate was recorded in Wad Al Helew locality in the south section (75%). In goats, the rate of BTV seropositivity was high (100%) in all localities (Table 2). There was a significant association (*p* ≤ 0.05) between seroprevalence of BTV infection and animal species, sex, and age. Thus, caprine species was more likely to be infected with BTV (100%, *p* ≤ 0.000) than ovine species, females were more infected than males (92.8% and 85.5%; *p* ≤ 0.003), and animals that aged 6–11 months were more likely to be infected with BTV (93.9% and 85.5%; *p* ≤ 0.000) compared to animals that aged 1 year and more. The infection with BTV was higher in the winter season (91.4%) (Table 3).

## 4. Discussion

Of late, BTV has become a major concern worldwide as well as the target of many epizootiological studies and surveillance programs. In fact, starting from 1998, the global distribution of BTV has changed significantly after its northwards encroachment into previously nonendemic areas [38], and outbreaks of BTV infection, especially among sheep, were observed in various parts of the world. However, in general, meager data are available about *Orbiviruses* in the country. Further studies on Sudanese BTV serogroup are thus required to delineate their distribution, ecology, biology, and molecular epidemiology.

This study was carried out to determine the prevalence of BTV antibodies and associated risk factors among domestic small ruminants (sheep and goats) in Kassala State, Eastern Sudan, where the infection has not been previously reported. In Sudan, an early serological survey showed that BT infection is generally widespread among all domestic ruminants [27]. Subsequent serological surveys for BTV group-specific antibodies further confirmed the widespread nature of the infection in various parts of the country [28, 29, 31, 36, 39]. The present study showed that the overall seroprevalence of BTV group-specific antibodies in sheep and goats in Kassala state was substantially higher than previously recorded in other regions of Sudan. The high prevalence rate of BTV antibodies in Kassala state may indicate favorable climatic condition for breeding and survival of various stages of *Culicoides* vectors in this region [40]. This is supported by the fact that the Dongla locality in the Northern state of Sudan was reported to be free of BTV infection mainly due to the very hot and dry climatic conditions which render the area unfavorable for the activity and maintenance of the life cycle of the insect vector [27]. In the current study, there was a significant association between the BTV seroprevalence rate and the animal age. This is in agreement with Mohammadi et al. [41], who recorded positive association of BTV infection and age. This is probably attributed to multiple exposures of older animals to infected *Culicoides* vectors. In addition, in our study area, young animals (6–11 months) are usually raised indoors and are well taken care of by the owners protecting them from contracting infectious diseases, particularly the vector-borne diseases.

In the current study, seropositivity to BTV in goats was significantly higher (*p* ≤ 0.000) than sheep. Higher seroprevalence among goats compared to sheep demonstrates that goats may play a crucial role in BTV epidemiology in Kassala state. However, sheep that are more susceptible animals to BT show clear clinical signs and die of the disease, and goats are more resistant and can survive with infection [42]. It is also a confirmed fact that goats with their minimum clinical manifestations keep higher titer of BTV and may be the potential source of infection to other susceptible animals [43]. The present study showed that females were more infected (*p* ≤ 0.003) than males with BTV. However, it is hard to explain that this might be attributed to a sample size bias originating from the availability of animals on the farms.

In the present study, the highest rates of BTV infection were recorded in Kassala and Western Kassala localities (100%), and the lowest rate was recorded in Wad Al Helew locality (75%), respectively. The high level of BTV infection in Kassala and Western Kassala localities (100%) may be attributed to the ecological factors in the rich savanna region that may favor higher density of the insect vector in these particular localities. In addition, Kassala state shares long international borders with Eritrea with no strict restriction on animals' movement across the borders which may allow introduction of infected animals into the localities. Similar high seroprevalence of BTV infection in various animal species was reported in several states in Sudan [28, 29, 31, 36, 39] and in countries around or near Sudan such as Libya (48.4%) [44]; Egypt (16.9%) [45]; Ethiopia (30.6%) [42]; and Saudi Arabia (47.3%) [46]. In addition, earlier epizootiological surveys also showed high prevalence rates for BTV seropositivity in Iran (93.5%) and Southern Turkey (88%) [47, 48].

Finally, it is worth noting that the BTV-specific antibodies recorded among ruminants in Kassala and other states in Sudan indicate natural infection as unlike some of the other countries; there is no vaccination program for control of the disease in the country. In addition, animal movement across the open borders can be another factor that affects BTV seropositivity results. Movement of infected animals would permit the local midge population to become infected with subsequent rise in BTV infection rates. However, this factor is not addressed in this study and should deserve further investigations. In addition, attempts to isolate and identify virus serotypes circulating in Kassala state as well as in other states of Eastern Sudan should be undertaken to facilitate further epidemiological investigations and to help formulate sound control programs.

To the best of our knowledge, this is the first study that estimates the prevalence and distribution of BTV antibodies and to detect virus activity in sheep and goats in localities of Kassala State, Eastern Sudan.

## 5. Conclusions

It could be concluded that BTV antibodies are highly prevalent in Kassala state and that susceptible livestock in eastern states are at risk of becoming severely infected with BTV. However, the specific BTV serotypes circulating in the region remain to be identified. In addition, investigations on the species of *Culicoides* vectors involved in the transmission of BTV, their biology, and ecology in the area should also be embarked upon to better predict and respond to BT disease in Kassala State, Sudan.

## Figures and Tables

**Table 1:** The overall seroprevalence of BTV in different geographic areas in Kassala state.

Section	Ecological characteristics	Total of tested	Positive (prevalence (%))	Chi square	df^*∗∗*^	*p* value
East	Savanna	260	238 (91.5)	17.102	5	0.004^*∗*^
West	Rich savanna	144	139 (96.5)			
North	Savanna	161	146 (90.7)			
N. East	Savanna	161	136 (84.5)			
South	Rich savanna	56	52 (92.9)			
Center	Rich savanna	27	27 (100)			

^*∗*^ *p* value ≤ 0.05 is significant. ^*∗∗*^df: degree of freedom.

**Table 2:** Influence of the locality risk factor on overall seroprevalence of BTV in Kassala state.

Risk factor	Overall results		Overall results	
	Sheep		Goats	
Locality	Total of tested	Positive	Total of tested	Positive
Rural Kassala	164	134 (81.7%)	149	149 (100%)
Kassala	25	25 (100%)	2	2 (100%)
Halfa	52	47 (90.4%)	32	32 (100%)
Western Kassala	32	32 (100%)	28	28 (100%)
Khashm Ghirba	58	48 (82.8%)	0	0
Wad Al Helew	28	21 (75%)	22	22 (100%)
Telkuk	28	24 (85.7)	28	28 (100%)
Aroma	51	41 (80.4%)	60	60 (100%)
Northern Delta	21	16 (76.2)	29	29 (100%)
Total	459	388 (84.5%)	350	350 (100%)

**Table 3:** Univariate and multivariate logistic regression analysis of selected risk factors associated with bluetongue virus in sheep and goats in Kassala state.

Risk factor		Results		Chi square	df	*p* value
		Positive	Negative			
Species	Caprine	350 (100%)	0	59.348	1	0.001^*∗*^
	Ovine	388 (84.5%)	71			

Sex	Female	591 (92.8%)	46	9.048	1	0.003^*∗*^
	Male	147 (85.5%)	25			

Age	6–11 months	527 (93.9%)	34	17.348	2	0.001^*∗*^
	1 year	189 (85.5%)	32			
	>1 year	22 (81.5%)	5			

Season	Rainy	451 (91.3%)	43	0.092	2	0.955
	Winter	172 (91.4) %)	16			
	Summer	115 (90 6%)	12			

^*∗*^ *p* value ≤ 0.05 is significant.

## Data Availability

The data used to support the findings of this study are available from the corresponding author upon request.
